# A Sensory Feedback Neural Stimulator Prototype for Both Implantable and Wearable Applications

**DOI:** 10.3390/mi15040480

**Published:** 2024-03-30

**Authors:** Federico Mereu, Francesca Cordella, Roberto Paolini, Alessia Scarpelli, Andrea Demofonti, Loredana Zollo, Emanuele Gruppioni

**Affiliations:** 1 Centro Protesi Inail, Vigorso di Budrio, 40054 Bologna, Italy; e.gruppioni@inail.it; 2 Unit of Advanced Robotics and Human-Centred Technologies, Università Campus Bio-Medico di Roma, 00128 Rome, Italy; f.cordella@unicampus.it (F.C.); r.paolini@unicampus.it (R.P.); a.scarpelli@unicampus.it (A.S.); a.demofonti@unicampus.it (A.D.); l.zollo@unicampus.it (L.Z.)

**Keywords:** sensory feedback, nerve stimulation, PNS, TENS, implantable electronics, wearable

## Abstract

The restoration of sensory feedback is one of the current challenges in the field of prosthetics. This work, following the analysis of the various types of sensory feedback, aims to present a prototype device that could be used both for implantable applications to perform PNS and for wearable applications, performing TENS, to restore sensory feedback. The two systems are composed of three electronic boards that are presented in detail, as well as the bench tests carried out. To the authors’ best knowledge, this work presents the first device that can be used in a dual scenario for restoring sensory feedback. Both the implantable and wearable versions respected the expected values regarding the stimulation parameters. In its implantable version, the proposed system allows simultaneous and independent stimulation of 30 channels. Furthermore, the capacity of the wearable version to elicit somatic sensations was evaluated on healthy participants demonstrating performance comparable with commercial solutions.

## 1. Introduction

Restoring the missing limb’s functionality is the primary goal in the field of limb prosthesis, especially in the upper limb. Two major areas can be distinguished in this regard: control and sensory feedback. These two domains align with the innate functions of the human hand, particularly in the efferent (control) and afferent (feedback) pathways.

If, on the side of control, it is possible to find numerous solutions (even rather consolidated), both commercial and in the field of research, that exploit different technologies to achieve the aim, on the side of restoring sensory feedback the challenge is still open. It significantly impacts not only the prosthesis functionality but also its embodiment by the users. The VINCENTevolution [1] (commercially available since the second edition but currently on the fourth) is the only commercial solution that enables the users to receive sensory feedback from the prosthesis. Nonetheless, a variety of stimulation approaches can be found in the literature, differing in terms of technology employed (electrodes, electronics, algorithms), level of invasiveness, and implemented stimulation nature. There is still no consensus on the most efficient way to restore the afferent channel, even though it is evident that some approaches are less effective than others.

As reported in [2], the ideal sensory feedback restoration device should elicit sensations that are comparable in quality and location to those experienced by the intact limb. This is known as homology, where the sensory neuroprosthesis elicits sensations that match the external stimulus’s quality, and somatotopy, where it elicits sensations that match the location. Moreover, these aspects maximize the effectiveness and acceptance of the stimulus [3]. The method employed for the stimulation has a significant impact on the feelings that are evoked. In particular, the elicited sensations are somatotopic and homologous since the signals are induced directly within the nerve.

According to the reviews [4,5] sensory feedback can be categorized into substitution, modality-matched, and somatotopically matched methods. Substitution uses a feedback signal that is not matched to the prosthesis stimulus, common methods include translating tactile information through vibration, electro-cutaneous, or auditory stimuli. Modality matching matches the stimulus but may not be physiologically representative; typically, tactile information is communicated using force or pressure. Somatotopic matching is anatomically matched to the stimulus’s location; targeted sensory reinnervation (TSR), peripheral nerve stimulation (PNS), and phantom mapping belong to this method. 

Technologies that restore non-somatotopic sensations, such as substitution and modality-matched feedback methods, have disadvantages; these include artifacts on the recording system used in myoelectric prosthesis control caused by stimulation, system miniaturization, power consumption, and the unpleasant sensation elicited. The fact that these technologies do not require any surgical intervention is a huge advantage, but the previously listed problems likely hinder their practical acceptance [2,6]. 

Although the three somatotopically matched feedback solutions appear to be better than substitution and modality-matched feedback regarding sensations perceived by the patient, they also have limitations.

The primary issue with phantom mapping is that amputees—especially those whose amputation was not recent—do not always experience phantom limb sensation, so this method has limitations mainly in terms of applicability.

Targeted sensory reinnervation (TSR) has a different problem: although it necessitates surgery and is therefore invasive, it would enable the use of non-invasive stimulation techniques because it reinnervates the neural pathways, originating in the hand, of a target area [7,8]. These pathways are useful for stimulation, but the patient perceives them as natural. Furthermore, since TSR is a relatively recent approach, it is not possible to say with certainty what its long-term effectiveness is.

**Figure 1:**
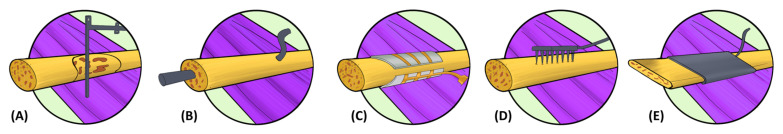
Different types of electrodes for peripheral nerve stimulation. (**A**) Transverse intrafascicular multichannel electrode—TIME; (**B**) longitudinal intra-fascicular electrode—LIFE; (**C**) Cuff; (**D**) Utah Slanted Electrode Array—USEA; and (**E**) flat interface nerve electrode—FINE.

Since the original afferent neural pathways remain intact proximally after upper limb amputation and can be utilized for prosthetic interface [9,10], peripheral nerve stimulation (PNS) appears to be the most viable treatment option. According to this theory, invasive neural electrodes have been used to strategically stimulate nerve afferents electrically to recreate natural physiological feedback [11,12]. These electrodes can interact with the nerve in various ways and come in a variety of materials and forms [2,5,13]. The most commonly used in PNS are:

- Transverse intra-fascicular multichannel electrodes (TIME)—intraneural, meaning they are implanted inside a peripheral nerve, enabling the transmission of information from the patient’s periphery to their nerves (Figure 1A).
- Longitudinal intra-fascicular electrode (LIFE)—intrafascicular, or placed inside a nerve beam, are used to stimulate specific nerve fibers (Figure 1B).
- Cuff—extraneural, meaning they encircle the nerve without entering it, making it possible to stimulate or capture a peripheral neuron activity. These electrodes have the shape of a shackle, hence their name (Figure 1C).
- Utah Slanted Electrode Array (USEA)—intra-fascicular electrodes, meaning they are placed inside a nerve beam and can be used to record or stimulate peripheral nerve activity (Figure 1D).
- Flat interface nerve electrode (FINE)—placed intraperitoneally, or inside a peripheral nerve, to record or stimulate peripheral nerve activity (Figure 1E).

However, PNS proves to be quite invasive, as it requires surgery and involves implanting electrodes and an electronic board, whose duration is unknown, within the patient [14,15].

In response to injury or illness, neural stimulators are implantable, active devices that electrically stimulate neural or neuro-muscular tissue to provide improved motor control, sensory feedback, or therapeutic intervention [16]. There are numerous solutions, both in the research [17,18,19,20] and commercial [21] fields, most of them used for the treatment of vagus nerve pain. In other studies, PNS was used for sensory feedback, and it was demonstrated that it is suitable for restoring tactile sensations, such as touch, pressure, and slippage, as well as proprioceptive sensations, such as position sense and movement [12,14,22] during grasping tasks, providing amputees with the capability of distinguishing between the size and firmness of various things [7,12]. However, these approaches include the implanted electrodes with the main stimulator outside the body. The device shape will vary depending on the intended implant location. The majority of neurostimulators have a recognizable flat, rounded appearance and are placed in the belly or subclavicular region [23].

With a weight of 17 g, a volume of 8 cm^3^, and dimensions of 48 mm × 29 mm × 7.5 mm, the NDI Medical Micropulse stimulator (MICROPULSE^®^, NDI Medical, Cleveland, OH, USA) is a completely programmable implantable pulse generator (IPG) [24]. This patented system, developed for the treatment of incontinence and pain, comes with a rechargeable battery whose life is estimated at 30 days, after which it can be recharged by applying the dedicated system to the implant.

Another solution presents an implantable nerve stimulator, used for different purposes: in [25], an implantable wireless multi-channel neural prosthesis for epidural stimulation was presented. The implantable board (46 mm × 42.8 mm × 8.8 mm) presents a receiving coil for the supply through wireless power transfer, and three stimulating systems, each providing six active channels and two return electrodes. In [26], a wireless peripheral nerve stimulation system, namely ReStore, was described. The implantable part of the system (13 mm × 8 mm × 3 mm) is able to perform nerve stimulation using two electrodes, delivering charge-balanced, biphasic current pulses of varying amplitudes, pulse widths, frequencies, and train durations. The system was in vivo tested and applied on large animals to study long-term biocompatibility. The solution used in [27] provides recordings from 128 channels and the stimulation by using two output channels, each one selectable through a multiplexer with a minimum switching time delay of 20 µs. The stimulation was a biphasic charge-balanced pulse, with an amplitude ranging from ±500 µA up to ±4 mA and a pulse width of 1 ms. The stimulation frequency used was 20 Hz for a stimulation duration of 10 s. The system with the lowest neutralization latency and power consumption, together with a programmable balancing precision and an innovative charge balancing technique, is the 24-channel neurostimulator IC described in [28]. Lastly, the strategy outlined in [29] includes the use of commercially accessible components in an implantable neurostimulation system for long-term behavioral and biological studies of neuropathic pain treatment.

THE PROPOSED APPROACH Given that peripheral nerve stimulation appears to be the most suitable option to restore sensory feedback, the aim of this study is to present the design of a neurostimulator system that could be used both in an implantable and wearable scenario. The system developed for the implantable scenario is composed of two printed circuit boards (PCBs): the first is mainly responsible for encoding the stimulation data and generating the wireless power transfer (WPT) for the power supply of the second board; the second PCB, implantable, is responsible for generating the stimulation patterns and managing the stimulation itself. The proposed implantable system allows stimulation on 30 independent active channels, each with a maximum pulse amplitude of ±5 mA. To make the system also adaptable to a completely external and wearable scenario it is necessary to add another PCB to the system. This PCB allows an increase in voltage compliance, mandatory to be used as an electrotactile feedback restoration solution (i.e., through transcutaneous electrical nerve stimulation—TENS); in this case, the system provides up to eight independent active channels. Bench tests were carried out to evaluate the output stimuli, comparing them with the theoretical expected values and with those obtained with a commercial bench stimulator, used as a reference. To the authors’ best knowledge, this is the first sensory feedback prototype that allows neural stimulation to be performed in two different application scenarios (implantable and wearable) using the same core.

## 2. Materials and Methods

One of the biggest challenges in the field of sensory feedback via PNS concerns the design of the implantable system; in fact, this must have low energy consumption, have reliable and stable long-term operation, and, obviously, respect the biocompatibility characteristics, have small dimensions and must be able to communicate with the outside. The stimulation waveform used in this study was the symmetric biphasic square wave, as used in the literature both for PNS [12,25,26,27,28,29] and for TENS [30,31,32,33,34,35] since it was shown to be able to elicit a more comfortable sensation among the other stimulation waveforms. The biphasic square wave settable parameters are the pulse amplitude (PA), the pulse width (PW), the pulse frequency (PF), and stimulation duration (i.e., the number of pulses).

### 2.1. System Architecture and Overview

The stimulation system consists of two major components: the external wearable transmitter (EWT) and the implantable pulse generator (IPG), described in detail in the following sections. During the functioning, the system receives data from the instrumented prosthesis through cabled controller area network (CAN) bus. These data were processed and codified into a string of parameters by the EWT that was sent to the IPG through Bluetooth. A reply from the IPG was always expected so that correct run-time functioning could be verified. The IPG was designed to be implanted in a subcutaneous area, to allow it to be powered via inductive coupling. Ideally, for patients with transradial and transhumeral amputations, it should be positioned on the stump; for patients with shoulder disarticulation, however, a subclavicular or chest positioning would be more appropriate. This would, in fact, allow the wearable transmitter to be worn easily: via an elastic band in the case of transradial or transhumeral amputation or in a special pocket in the case of a patient with shoulder disarticulation. The IPG was powered through an inductive coupling by the wearable transmitter; both the primary and the secondary coils used in the wearable transmitter and in the IPG, respectively, present a magnet in their center for mutual alignment, to obtain higher efficiency for the WPT.

**Figure 2:**
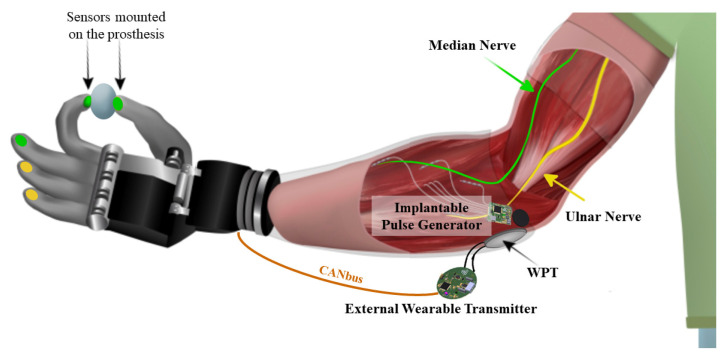
Representation of the proposed system.

Previous studies [12,36,37] have shown that the PNS used for the restitution of sensory feedback has the median and ulnar nerves as target nerves. Specifically, ulnar nerve stimulation restores touch perception in the ring and little fingers and medial region of the palm, while stimulation of the median nerve restores touch perception in the thumb–index–middle finger and the lateral part of the palm. Figure 2 shows an example of application of the prototype in its implantable version, in the specific case of transradial amputation, identifying ulnar and median nerves as the stimulation target. 

**Figure 3:**
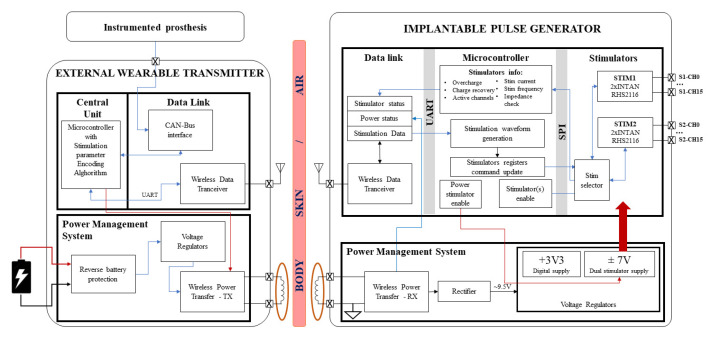
System block diagram.

**Figure 4:**
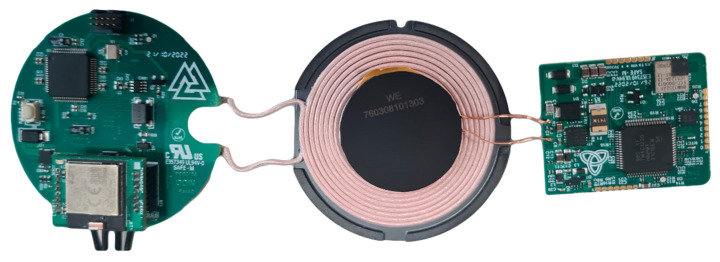
Implantable version of the system, composed of two boards: EWT (**left**) and IPG (**right**).

The operation in the implantable scenario can be schematized as in Figure 3, in which it is possible to observe the functional blocks of the EWT and IPG, while the implantable system boards are shown in Figure 4.

Since the manipulation gestures mostly involve the use of the thumb and index finger, it was decided to divide the thirty active channels available into three groups of ten, to use two electrodes on the median nerve and one on the ulnar nerve. Furthermore, four more contacts were added to each of the three electrodes to be used as ground/reference.

**Figure 5:**
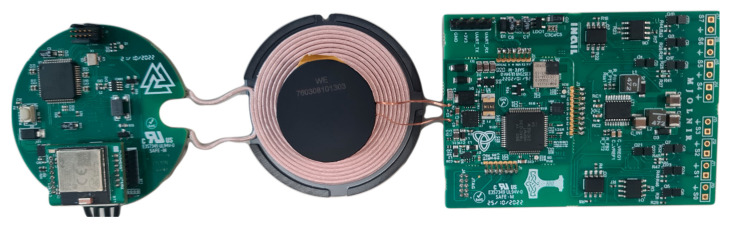
Wearable version of the system, composed of three boards: EWT (**left**) and IPG mounted on the add-on board (**right**).

Adding the third PCB to the implantable system, it is possible to adapt the system to a completely external and wearable scenario. The voltage compliance of the implantable system (14 V) is insufficient for the wearable scenario: as discussed in [38,39], high voltage compliance is necessary to adapt to variations in electrode–skin impedance, allowing the device to stimulate loads over 10 kΩ with currents of 5 mA. So, for external stimulation (i.e., TENS), it is mandatory to have a higher voltage compliance, which is achieved by adding the third board. Additionally, the quantity of stimulation channels required for external applications is less than that required for PNS; as reported in the literature, TENS was already used for sensory feedback with a number of stimulating channels ranging between 2 and 4 [40,41,42,43]. For this reason, the third board was developed with a lower number of active channels. To work properly, the IPG must be mounted on the add-on board as shown in Figure 5.

### 2.2. External Wearable Transmitter 

The EWT was mainly composed of a 4-layer round PCB, with a diameter of 50 mm and a thickness of 1.6 mm (excluding components) and the transmitter coil (Würth Elektronik eiSos GmbH & Co. KG, Munich, Deutschland—manufacturer ID: 760308100110), a 24 µH coil, with a diameter of 50 mm. There were 3 macroblocks: power block, communication block, and the central unit block. 

In the power block, the reverse battery protection circuit with p-channel MOSFET [44] (ON Semiconductor, Phoenix, AZ, USA—manufacturer ID: FDS6681Z), the overcurrent/short-circuit protection through a fuse, and the transmitter part of the WPT circuit were implemented. Finally, the rest of the circuit was supplied at 3.3 volt through a switching voltage regulator (ST Microelectronics, Geneva, Switzerland—manufacturer ID: L7986TR). 

The communication block includes the CAN bus and the Bluetooth parts. The first one was used to communicate data with a previously used prosthetic device [45], exchanging data with a data rate of 1 Mbit/s. The Bluetooth part was composed of the BGX13P (Silicon Labs, Austin, TX, USA—manufacturer ID: BGX13P22GA-V21R), a Bluetooth low energy (BLE) module with a built-in antenna that integrates a Bluetooth 5.0 compliant protocol. This module allows communication with the IPG, with a frequency band of 2.4 GHz; data were exchanged between the module and the microcontroller through UART protocol, with the 8N1 configuration and a baud-rate equal to 115,200 bps.

The core of the central unit block is the 32-bit Arm Cortex-M4F-based MCU TM4C123GH6PM (Texas Instruments, Dallas, TX, USA—manufacturer ID: TM4C123GH6PMI). This microcontroller, programmed in C language with Code Composer Studio IDE v10.1.0, was responsible for the following tasks:

- Receiving grasping force data from the sensorized prosthesis.
- Encoding the stimulation parameters using an ad hoc algorithm.
- Sending the stimulation parameters to the IPG via Bluetooth.
- Continuously monitoring the WPT and IPG’s status.

Firstly, the system is initialized and, after the connection with the prosthetic system is confirmed, the WPT circuit is activated. The system enters a loop where it monitors the status of the IPG and the WPT, awaiting data from the prosthesis. Upon receiving this data, stimulation parameters are encoded and sent to the IPG via Bluetooth. Feedback from the IPG is monitored to ensure successful stimulation. If successful, the loop continues; if not, the system investigates the issue. If the IPG can resolve the problem, the error is saved as a warning and normal functioning continues. If the IPG fails to provide feedback within a time limit or the issue cannot be resolved, the WPT is disabled, effectively turning off the IPG.

### 2.3. Implantable Pulse Generator

The IPG comprises a receiver coil (Würth Elektronik eiSos GmbH & Co. KG, Munich, Deutschland—manufacturer ID: 760308101303), a 47 µH coil, with a diameter of 26.3 mm, and a 6-layer PCB, 33.5 mm × 27.5 mm and a thickness of 3.3 mm (1 mm excluding components). The board presents 4 macroblocks: the power management block, the communication block, the central unit block, and the stimulator block. 

**Figure 6:**
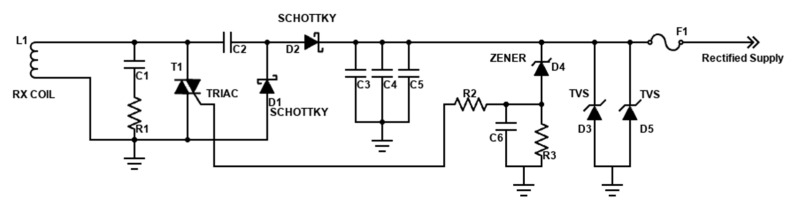
Wireless power receiving and rectification circuit.

In the power management block, there are two parts: the first one (Figure 6) is the receiving circuit of the wireless power transfer that generates rectified power when the voltage applied to the receiver coil exceeds a threshold; the output voltage is 9.5~10 V while the maximum output current is 750 mA (with maximum distance between transmitter and receiver coil equal to 15 mm).

The second part consists of two voltage regulators, one to supply the digital part with fixed 3.3 V (ROHM Semiconductor, Ukyo Ward, Kyoto, Japan—manufacturer ID: BD9P135MUF-CE2), and one to generate the dual power supply ±7 V (ROHM Semiconductor, Ukyo Ward, Kyoto, Japan—manufacturer ID: BD8316GWL-E2) needed for nerve stimulation.

The communication block comprehends a BGX13S (Silicon Labs, Austin, TX, USA—manufacturer ID: BGX13S22GA-V31R), a Bluetooth IC with a built-in antenna, which mounts the same chipset of the BGX13P. As for the wearable transmitter, in the IPG data are exchanged with the microcontroller through UART protocol, with the standard 8N1 configuration and a baud rate equal to 115,200 bps.

Another TM4C123GH6PM was mounted in the central block unit of the IPG in order to process the received stimulation parameters to commands for the stimulator chips to generate the desired waveform, and to monitor the wireless power receiver and stimulator chips’ status. 

**Figure 7:**
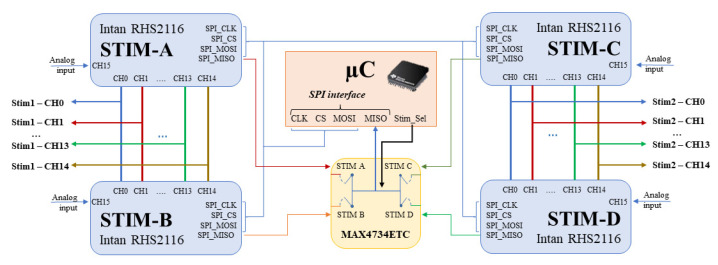
Microcontroller stimulator configuration.

The stimulator block was composed of four RHS2116 (INTAN Technologies, Los Angeles, CA, USA—manufacturer ID: RHS2116) stimulator chips. With 16 independent stimulator/amplifier channels, the RHS2116 stim/amplifier chip is a comprehensive bidirectional electrophysiological interface. A programmable constant current stimulator and a tunable low-noise biopotential amplifier that may produce stimulation pulses for extracellular microelectrodes were included in each channel. This chip comprehends stimulators, charge recovery circuits, amplifiers, filters, a 16-bit ADC, and impedance measurement. Stimulators source and sink currents ranging from ±10 nA to ±2.55 mA over a 14 V range with integrated compliance monitors. For this reason, to provide a stimulation current up to ±5 mA, it was decided to use the chips in a coupled manner, connecting the channels of two chips with the same position (i.e., StimA Ch0 with StimB Ch0 and so on) as shown in Figure 7.

In this way, each couple provides up to 15 stimulation channels (1 channel per chip was reserved for acquiring application), for a total of 30 stimulation and 4 acquiring channels. Regarding the connection with the electrodes, the prototype was designed not to be limited to the type of electrode but rather features three connectors (Polarized Nano PZN-14-AA, NanoStrip Series-OMNETICS, Minneapolis, MN, USA) with 14 contacts (10 for each of the active channels + 4 for reference/GND). In this way, given that it is possible to ask the supplier to customize the electrode connector based on the position and number of contacts desired for the specific application, the system is potentially compatible with all electrodes.

The stimulators communicate with the microcontroller via SPI (packet size = 32-bit data words frequency = 20 MHz) and always function as the SPI slave device of the architecture. The MOSI and CLK lines are common to all four chips, while, two CS lines were used, each of which is shared by the chips belonging to the same pair couple. This was because the microcontroller needed to simultaneously send the same data to every chip in the same pair. To read the status from each of the four chips, the microcontroller’s MISO line is connected to the common of a selector (MAX4734ETC-Analog Devices, Wilmington, MA, USA), which is short-circuited with one of the four MISO lines originating from the stimulator’s chips via the dedicated line Stim_Sel. In this way, it was possible to use only one SPI-Bus peripheral of the microcontroller. 

To summarize, the IPG controls stimulation chips, with an initialization procedure upon activation and connection with the EWT. If connection fails, IPG goes into standby. If successful, stimulation chips and managers are initialized. The microcontroller reads chip status and sends feedback to EWT. Instructions follow for pair selection, channel activation, parameter values, status monitoring, and checks. Positive feedback follows successful stimulation; error-related messages are sent otherwise. Errors are categorized as low, medium, or high level, with corresponding resolution procedures. Low-level errors allow for error resolution procedures, medium-level errors involve shutting down stimulation chips, and high-level errors disable stimulators and wait for procedures. The worst scenario involves turning off the entire IPG if necessary.

### 2.4. Add-On for Wearable Application

**Figure 8:**
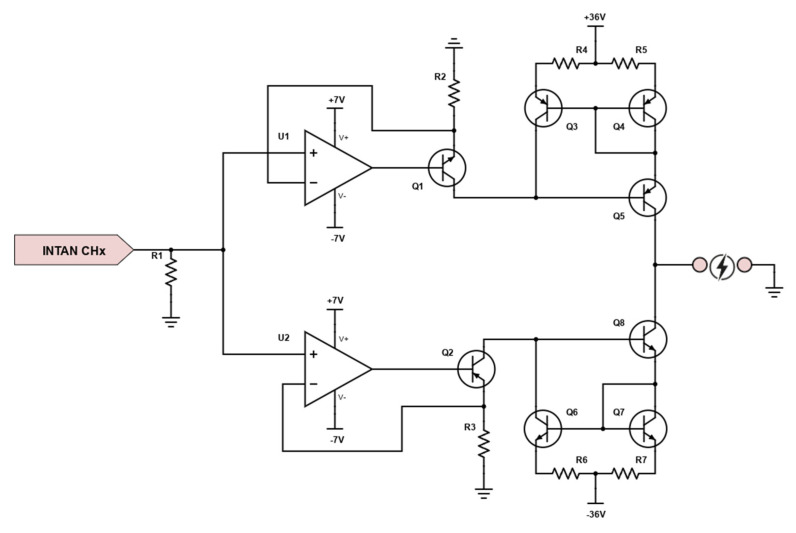
Circuit diagram of the current mirror used to adapt the voltage compliance of the stimulation signal.

The technology that was previously described has a restricted voltage compliance (±7 V) that is appropriate for PNS applications [29,46] but insufficient for external stimulation. The system must have one more PCB added to overcome this limitation. Furthermore, although it is possible to be more selective with invasive stimulation by stimulating more sites, selectivity decreases with TENS; for this reason, just 8 channels—all of which are part of the StimA-B pair—were chosen. In this PCB, there are two blocks: the voltage compliance adapter block and the current mirror block. The first one is related to the step-up voltage circuit, obtained by using a DC-to-DC switching voltage regulator with independent positive and negative outputs, namely ADP5071 (Analog Devices, Wilmington, MA, USA—manufacturer ID: ADP5071AREZ) to have a voltage compliance of 72 V (±36 V). The second block comprehends two Wilson current mirrors for each channel, one for the anodic phase of the square wave (i.e., the positive part) and one for the cathodic phase (i.e., the negative part) as shown in Figure 8.

For each current mirror, U1 and U2 are rail-to-rail OpAmps, Q1-Q6-Q7-Q8 are NPN BJTs, and Q2-Q3-Q4-Q5 are PNP BJTs.

To summarize, the stimulator is a modular system, composed of two or three boards based on the application scenario. The technical characteristics of the three boards are reported in Table 1. The parts common to the two systems are the EWT and the IPG. The EWT is essentially responsible for powering the entire system and encoding the stimulation parameters starting from the force information received from a sensorized prosthesis. The “heart” of the system, common to both versions, is the IPG. This PCB allows neural stimulation to be carried out on 30 active channels, thanks to the use of 4 RHS2116 chips. Regarding the wearable version, it is necessary to add the third board to increase the voltage compliance of the system, making it suitable for TENS, and maintaining control of the current stimulation via the current mirrors. All this, however, resulted in a reduction in the stimulation channels from 30 to 8, which are still suitable for TENS [40,41,42,43].

### 2.5. Bench Measurement Setup

The following bench tests were performed to confirm that both system versions were operating properly. To evaluate whether the current values set for stimulation were consistent with the expected ones, the oscilloscope probe was connected to the ends of a 1 kΩ resistor, which was connected to the selected stimulation channel; additionally, data were recorded, through an oscilloscope, for each test. The following parameters have been tested for the implanted version:

- PA values: starting from the minimum of 1 µA up to 2.5 mA with steps of 100 µA.
- PW values: starting from the minimum of 100 µs up to 500 µs with steps of 100 µs.
- PF values: starting from the minimum of 50 Hz up to 500 Hz with steps of 50 Hz.

Once the correctness of the values had been verified, PF and PW were set to their minimum values, 50 Hz and 100 µs, respectively, as completed in [32], and, for each of the PA values, the stimulation waveforms were acquired three times.

The following parameters have been tested for the TENS version:

- PA values: starting from the minimum of 1 mA up to 5 mA with steps of 500 µA.
- PW values: starting from the minimum of 100 µs up to 500 µs with steps of 100 µs.
- PF values: starting from the minimum of 50 Hz up to 500 Hz with steps of 50 Hz.

Once the correctness of the values has been verified, as for the implantable version, PF and PW were set to 50 Hz and 100 µs, respectively, and, for each of the PA values, the stimulation waveforms were acquired three times.

Having verified correct functioning, the next step was to characterize the stimulator in its TENS version using resistors with standard values (4.7 kΩ–5.6 kΩ–6.8 kΩ–10 kΩ–12 kΩ–15 kΩ–22 kΩ–27 kΩ–33 kΩ), which allow the entire range of voltage compliance to be evaluated (since with 1 mA and 33 kΩ the expected voltage drop is equal to 33 V), and it was verified that the voltage drop across the resistors was adequate/proportional to the theoretical expected voltage drop, according to the voltage compliance of the device. In this stage, a PA with values ranging from 1 mA to 5 mA, with steps of 0.5 mA, was employed, and the minimum values of PF and PW were set at 50 Hz and 100 µs, respectively. In parallel, a performance comparison was carried out with the benchtop stimulator STG4008 (Multi Channel Systems MCS, Reutlingen, Germany) with the same waveform, to verify any difference with a commercial bench stimulator used in previous studies. At this stage, since the STG4008 stimulator has a voltage compliance of 120 V, stimulation tests outside the voltage compliance range of the system presented in this work were not considered. Each stimulation waveform was acquired three times.

Additionally, the feasibility of conducting simultaneous stimulation on multiple channels with different stimulation parameters was verified; in particular, it was verified that eight channels could be used concurrently for each of the three electrodes by selecting a desired number of channels and applying distinct waveforms (i.e., different stimulation parameters) on each electrode. The stimulator can output up to six distinct waveforms simultaneously in this manner, two for each electrode. 

### 2.6. Mapping Protocol for TENS Version

The TENS version of the system was used to replicate the tests reported in [32]: (i) the mapping of the evoked sensations and (ii) the generation of the sensation. The aim is to confirm that the sensation experienced by the subjects was qualitatively equivalent independently of the adopted stimulator.

There are two phases to this process, in the first one the developed TENS stimulator was compared with the STG4008 benchtop stimulator to elicit somatic sensations in four healthy participants, aged between 27 and 43, (3 males and 1 female). The first phase aimed to evaluate both the minimum and the maximum current amplitude tolerable by each participant. A pair of commercial auto adhesive, circular, with a diameter of 25 mm, superficial electrodes (TensCare) was attached to the subjects’ forearms while they were comfortably seated, with their arms extended on a table. An oscilloscope was used to acquire the stimulation signals, in parallel with the electrodes. In this phase, the subjects were blindfolded. Each subject was stimulated by one of the two systems in a random order. Every PA value (ranging from 1 mA to 5 mA with steps of 0.5 mA) was used for every system, while PF and PW were set to 500 Hz and 500 µs, respectively. Following each stimulation, participants were asked to rate how intensely they felt the stimulation, from 0 (no perception) to 10 (highest intensity/pain). The maximum PA value was set equal to the one before a muscle contraction. It was feasible to deduce from the results of this phase, which are detailed in Section 3.2, that the two systems are equivalent as long as the voltage compliance required for stimulation is within the ±36 V range (i.e., the voltage compliance of the proposed stimulator). 

For the second phase, 7 healthy subjects (4 males and 3 females), aged between 22 and 31 years volunteered to participate. All subjects had no known neurological disorders and no previous experiences with TENS. The aim of the second phase was to qualitatively evaluate the sensation perceived by the subjects when the stimulation parameters changed. After positioning his or her left arm on a table and sitting comfortably in a chair, the subject’s targeted skin area was cleansed with alcohol. To specifically stimulate the subject’s nerves, four commercial auto adhesive, circular, superficial electrodes (TensCare) with a diameter of 25 mm were attached to the subject’s epidermidis; an oscilloscope was used to acquire the stimulation signals, in parallel with the electrodes. The optimal positioning was identified by varying the location of each pair of electrodes. During the phases of median and ulnar mapping, the PW and PF parameters were modulated, and the perceived sensations were recorded as follows.

The participant was asked to describe the sensation’s profundity, naturalness, intensity, and level of pain for each experiment. A five-point rating system was used to determine how natural the sensation was; the lowest number indicated an artificial sensation: natural, almost natural, perhaps natural, almost unnatural, and unnatural. Selecting between superficial, deep, or both allowed for the assessment of depth. Touch/pressure, vibration, tugging, tingling, pinching, scorching, cold, hot, wrist flexion, wrist extension, finger flexion, finger extension, and nothing were the options used to evaluate the quality. A scale from 0 to 10 was used to report the sensation’s pain and/or intensity. The maximum PA is set as the value below the motor threshold at which the subject reported a well-defined and conformable sensation.

## 3. Results

### 3.1. Bench Tests Results

With an error of less than 0.5%, the results obtained during the tests on the stimulation system for both the implantable and TENS versions seem to be consistent with the predicted theoretical values for PA, PW, and PF.

Regarding the implantable version, the tests demonstrate that the voltage drops across the 1 kΩ seem to be consistent with the predicted theoretical ones for all the PA, PW, and PF values (differences < 0.5%). Specifically, for PA there was a difference of 0.45%, while for PW and PF the difference was approximately 0.01%.

**Figure 9:**
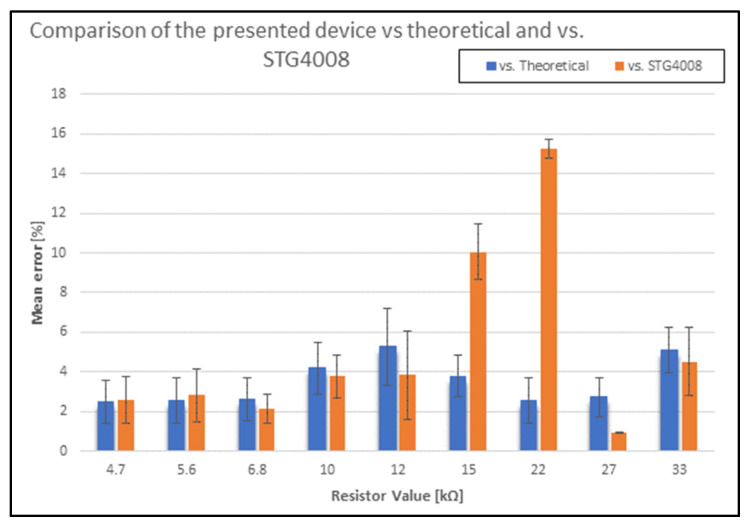
Average error, in percentage, between the presented system and the theoretical value (blue) and the bench stimulator STG4008 (orange), calculated over 3 repetitions.

Regarding the TENS stimulation system characterization, the collected data have demonstrated that the stimulator exhibits a performance that is consistent with theoretical ones (excluding tests outside of voltage compliance). The difference, in terms of mean error between the presented device and the theoretical values and the STG4008, is shown in Figure 9.

The average difference between the voltage drop measured across the resistors used and the theoretical one is 2.5% for R = 4.7 kΩ, 2.6% for R = 5.6 kΩ, 2.9% for R = 6.8 kΩ, 4.6% for R = 10 kΩ, 5.1% for R = 12 kΩ, 7% for R = 15 kΩ, and 10% for R = 22 kΩ. On the other hand, the voltage drop measured at the ends of the 27 kΩ and 33 kΩ resistors, using only PA = 1 mA, differs from the theoretical one by 2.75% and 5.11%, respectively.

The same tests were performed by comparing the voltage values read with those obtained using the STG4008 stimulator. The results showed that the voltage drop measured across the 27 kΩ and 33 kΩ resistors differed by 0.9% and 4.5%, respectively, and that the difference was less than 4% with the 4.7 kΩ, 5.6 kΩ, 6.8 kΩ, 10 kΩ, and 12 kΩ resistors, and 10.05% for the tests conducted with R = 15 kΩ and 15.2% with R = 22 kΩ.

To verify the simultaneity of multi-channel stimulation, the following stimulation parameters were used on two different channels. 

Channel 1–PA = 3 mA; PW = 300 us; PF = 400 Hz; duration = 500 ms. 

Channel 2–PA= 3.5 mA; PW = 400 us; PF = 500 Hz; duration = 500 ms.

**Figure 10:**
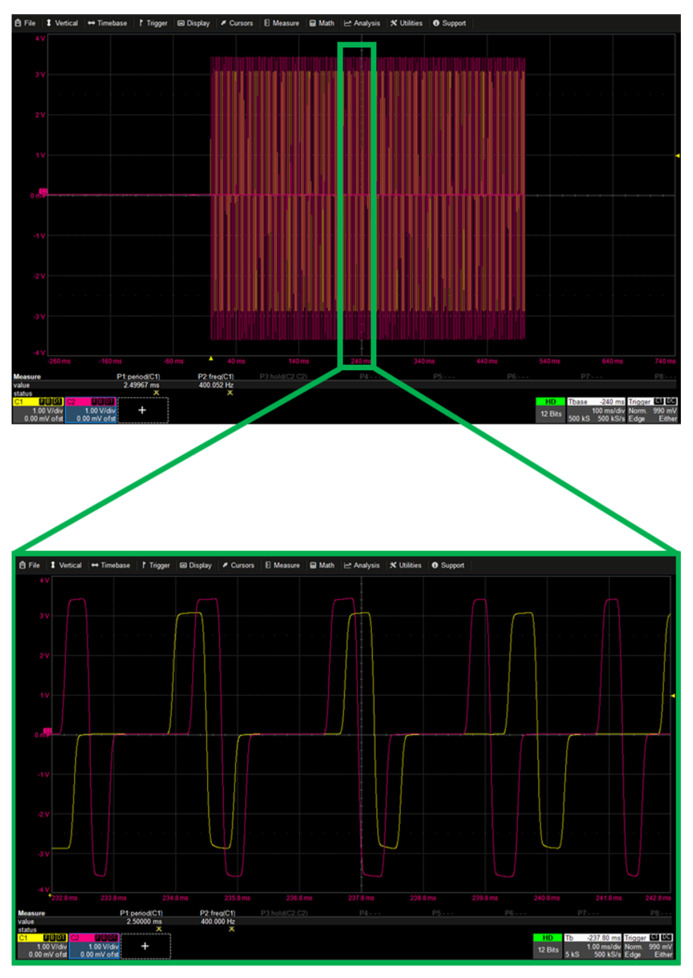
Multi-channel simultaneous stimulation, top: the 500 ms trace; bottom: zoom on a random section lasting 10 ms. Channel 1 is yellow, channel 2 is red.

The measurement was completed using a 1 kΩ resistor on each of the channels, obtaining the data with the oscilloscope (Figure 10).

To summarize, the results of the bench tests demonstrated the behavior and effectiveness of the proposed stimulation prototype, in both versions. Specifically, the results showed that there was a discrepancy of less than 0.5% between the recorded stimulation and the theoretical value for the implantable version, which is within the tolerance of the resistance used in the tests (1%). For the TENS version, the feasibility of stimulating using every proposed parameter combination was confirmed, highlighting the variations between the theoretical values and the values acquired using a commercial benchtop stimulator that has already been employed in previous studies.

### 3.2. Mapping Protocol Results

**Figure 11:**
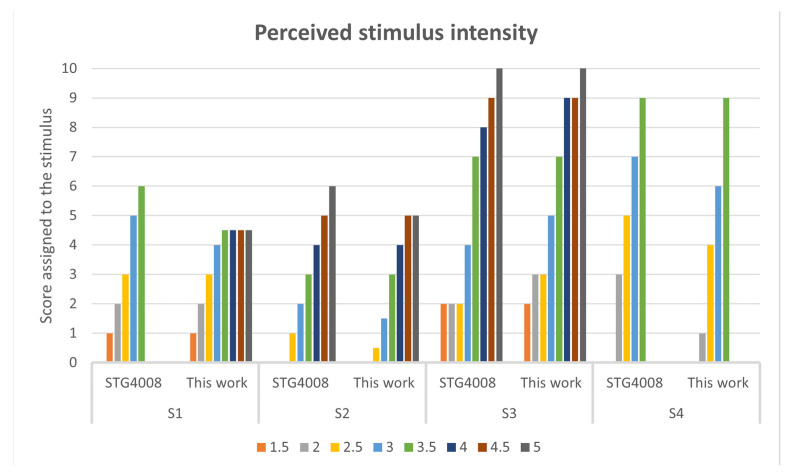
Perceived stimulus intensity, subdivided by subject and stimulator used.

The subjects participating in the first phase of the experiment reported perceiving rather similar sensations (Figure 11), in terms of intensity, for the stimulations carried out with the two systems until the voltage compliance necessary for the stimulation was less than ±36 V.

Using a PA of 1 mA, no subjects reported any type of sensation, and S2 did not perceive anything even when using 1.5 mA. Specifically, the minimum pulse amplitude value on average among the subjects was 1.625 ± 0.21, while the maximum one was 4.125 ± 0.415 mA. For subject S1, using the stimulator proposed in this work, saturation was detected using a PA of 3.5 mA while using the STG4008, a contraction was induced at the same PA; for S2 and S3 no saturation or contraction was detected, while for S4, using both stimulation systems, no PA values higher than 3.5 were used as muscle twitch was detected. 

The stimulus intensity levels perceived by the subjects are quite similar; the differences are as follows: for S1 using a PA equal to 3 and 3.5 mA, i.e., in the saturation zone of the proposed system, the intensity perceived with the proposed stimulator was lower by one point with respect to the STG4008; also for S2, using a PA equal to 5 mA, the intensity of the stimulus was perceived one point lower, for S3 the differences were detected using a PA equal to 2, 2.5, 3 and 4 mA, but the perceived intensity was one point higher using the proposed system; while for S4 the stimulations carried out with the proposed stimulator were perceived with a lower intensity level than those in which the commercial stimulator was used, except for the PA value equal to 3.5 mA.

For the second phase, both minimum and maximum current amplitude delivered to the participants was specific for each subject. The minimum PA mean value ± SD among the subjects is 1.8 ± 0.54 mA for the median nerve and 1.78 ± 0.39 mA for the ulnar nerve, while the maximum PA mean value ± SD among the subjects is 3.02 ± 0.67 mA for the median nerve and 3.19 ± 0.69 mA for the ulnar nerve.

All seven subjects reported being able to feel some sensations, for both the ulnar and median nerves. The most reported sensations were tingling, vibration, and pinch. 

For the median nerve, the stimulation was perceived as natural by four subjects (S1, S3, S6, S7) and almost natural for the other three. The sensations perceived were vibration (S1, S2, S5), pinch (S3, S4) and tingling (S6, S7). For the ulnar nerve, the stimulation was perceived as natural for two subjects (S6, S7), almost natural by S4, and perhaps natural for three subjects (S2, S3, S5); only S1 reported an unnatural sensation. The sensations perceived were vibration (S1, S2, S3, S7) and tingling (S4, S6).

Some exceptions are, in particular: S5 was the only one to feel the touch sensation, only for the ulnar nerve, while S7 reported a cold sensation in the index during the PF modulation (using 50 and 100 Hz, with 3.5 mA).

## 4. Discussion

The system presented in this work is a stimulator prototype designed to be suitable for both implantable and wearable applications. Precisely, in the implantable version, the system is composed of two boards (EWT and IPG) and has thirty active channels, divided into three groups of ten. For each group, the system is able to simultaneously generate up to three biphasic waveforms with different parameters, with a selectable number of channels. The voltage compliance is 14 V (±7 V), and, thanks to the coupling of the channels of the pairs of INTAN chips, the maximum pulse amplitude obtained with this system is 5 mA. The other stimulation parameter ranges are 100–600 Hz for the pulse frequency and 100–500 µs for the pulse width. Comparing the proposed IPG with the literature (Table 2), we propose a system equipped with a greater number of channels, 12 more than those reported in [25] and an order of magnitude greater than other works found in the literature [26,27,29]. Furthermore, the stimulus’s maximum PA is 1 mA greater than [27], 2 mA greater than [25], 3 mA greater than [29], and nearly 4 mA greater than [26]. Regarding the minimum amplitude, even if a PA lower than 1µA was not used in the current work (it is still possible to arrive at the order of nA) this appears to be an order of size smaller than [25,26,29] and 500 times smaller than [27]. The stimulus’s duration and frequency in the current work are quite similar to those used in all the works cited. The voltage compliance of 14 V is consistent with that achieved in [25,26,27] but is equal to half of that reported in [29]. Regarding the dimensions, however, the IPG presented in this work is smaller than the one used in [25] but larger than the one presented in [26], however, this difference can be justified by the presence of a number of channels significantly higher. 

As in [25,26], the IPG is wirelessly powered by an external board (the EWT) and contains no battery, thus eliminating the need for additional surgeries for battery replacement. 

The IPG was not further tested, but it was planned to carry out in vitro tests, once encapsulated in medical silicone or biocompatible epoxy resin, already used in invasive applications. Following the in vitro tests, it will be necessary to carry out tests on animals, subject to approval by the competent authorities. One of the limitations of the present work is that biocompatibility tests were not performed; however, biocompatibility tests have already been conducted on the materials composing the electrodes and the encapsulation of the stimulator, i.e., DC-734/platinum/polyamide. The biocompatibility tests have been performed on five New Zealand rabbits under the norm UNI EN ISO 10993-6:2017 Annex E [47]. The comparison with the control material, i.e., MED6015, corresponds to “minimal or no reaction”, proving that the selected materials are suitable for application in the implantable scenario. 

In addition to the biocompatibility discussed above, stability over the short, medium, and long periods has not been tested; nevertheless, risks associated with the implantable system can be identified. First of all, there are the risks associated with the surgery needed to implant the system. It is not possible to claim that the patient will experience the same sensations in the short and long term even if the operation is successful and the system operates as intended. One explanation could be that the electrode–nerve impedance may change over time as a result of changes in the nearby tissues. Another limitation is that the system will require additional surgery to be removed. However, it is possible to discuss the potential of the presented system. One of the advantages of the proposed system is the possible compatibility with different types of electrodes; this means that it is possible to choose which electrode to use based on the specific application. In fact, it is useful to specify that the quantity of charge transferred to the nerve must be evaluated for the specific case, also based on the nerve size and the nerve-electrode contact surface. For example, the use of cuff electrodes (Ardiem Medical, East Indiana, PA, USA) allows a theoretical contact given by the sum of the 16 contacts, each measuring 2.5 mm × 0.5 mm. Using instead an intraneural electrode such as the ds-FILE (Double-Sided FILament Electrode, Fraunhofer-IBMT, Saarbrücken, Sulzbach/Saar, Germany), usable both as TIME and as LIFE, the electrode–nerve contact surface would be given by the 16 active contacts, each with a surface equal to 150 μm × 50 μm, and the 2 reference/ground contacts, each with a surface equal to 200 μm × 2000 μm. The choice of the stimulus (i.e., the stimulation parameters) also depends on the technical specifications of the electrode: for the cuff electrodes it is possible to use currents in the order of mA, per contact, while for ds-FILE it is 40 μA per contact; however, the stimulation duration is set to a maximum of 0.5 s.

It has been observed in the literature that the restoration of sensory feedback is crucial for the incorporation of the prosthesis. As reported in [48], compared to a control group of healthy subjects, the amputee subject who used neural stimulation improved the reaction time for visual–tactile recognition. The increasingly common correlation between multisensory integration and distance from the body supports the idea that intraneural stimulation can bring a clear advantage over the embodiment of prostheses. Furthermore, it has been demonstrated in [12] that the presence of sensory feedback leads to an improvement also in prosthesis control since it increases patient–device confidence through loop closure. The commercial bench stimulator STG4008 (STG4008, Multichannel System MCS GmbH, Reutlingen, Germany) was used in these studies [12,48,49,50,51,52], and it was used as a reference in the present work demonstrating, from the bench tests, that the behavior of the two systems is comparable. Since the results obtained with the proposed prototype and the ones obtained with the STG4008 were comparable (as outlined in Section 3), the proposed device can be considered as effective as the commercial counterpart in stimulating single or multiple nerves, both invasively and not invasively.

For further stability in power transmission, a case for the EWT has been designed that facilitates wearability.

Adding the third board, the developed system can be used also for the wearable scenario for the TENS application. However, in this case, the number of channels is reduced to eight, maintaining the same ranges for the PA, PW, and PF parameters, but with an increase in voltage compliance to 72 V, which is mandatory to be used as an electrotactile feedback restoration solution. To the authors’ best knowledge, this is the first system that is suitable for both implantable and wearable applications. The proposed approach was compared with a commercial bench stimulator (STG4008), used in previous studies [12,32], to demonstrate the applicability for sensory restoration by TENS. Even if the voltage compliance is quite lower than the one of the STG4008 (~40% lower) since the goal was not to induce a muscle contraction but to replicate a touch sensation perceptible from the nerve, the data obtained with the bench tests are promising for the application of the system on humans. Thus, the system was used to verify the hypothesis that it is possible to replicate sensation in humans through TENS. The same procedure used in [32] was applied in this work, and the results confirmed the hypothesis. The results obtained on the four participants enrolled in the first phase demonstrated the capacity of the system to elicit sensations comparable to the one elicited with the commercial stimulator STG4008. In fact, for the same subject, the minimum values of PA, PW, and PF are the same regardless of the stimulation system used. It demonstrates that the proposed system is a valuable alternative to non-wearable commercial solutions.

The mean value of PA obtained during the second phase of the validation on healthy subjects was within the same range obtained in [32] for both the median and ulnar nerves. All the subjects were able to feel sensations due to the stimulation.

## 5. Conclusions

A new stimulator was presented in this work, which is suitable for both implantable and wearable applications. The prototype for the implantable version comprehends two electronic boards, the EWT and the IPG, and is able to generate up to 30 waveforms simultaneously. For the wearable scenario, the system requires an additional board, that allows for replicating the stimulation waveform by adapting the voltage compliance through a Wilson’s current mirror system. In this configuration, the proposed stimulator was employed to stimulate healthy subjects who reported feeling sensation, demonstrating the applicability of the system for restoring sensation. Prospects are to carry out tests on amputees, with the aim of returning sensory feedback following the information obtained from the prosthetic system. If promising results were to be obtained in this phase, a further development would be to integrate the stimulation system with the prosthetic system.

The procedure is substantially different for the implanted version, requiring numerous steps and more rigorous testing before human testing can begin. In fact, after choosing the electrodes to use, the implantable part of the system will be encapsulated inside a biocompatible material, specific for this type of application (i.e., medical silicone). The next phase involves carrying out a new test to verify the correct functioning of the system, verifying that the stimuli generated are consistent with the set parameters. Further tests concern the stability of the system, the heat generated by the device during stimulation, and the verification of fault systems, both hardware and firmware. Moreover, since it is still in the prototype phase, it is not possible to make further speculations on the usability of the system, also because each patient has different stump characteristics. However, the results presented in this work, obtained during the prototype tests, seem promising, but further development and testing are useful and necessary. 

## Figures and Tables

**Table 1:** Technical characteristics of the system’s boards.

	EWT	IPG	Add-On
Dimension [mm]	Ø 50 × 1.6	33.5 × 27.5 × 1	70 × 50 × 1.6
PCB stack layout	4 layers	6 layers	4 layers
Power supply	12 V	9.5~10 V	9.5~10 V
Components	105	108	130
Stimulation channels	/	30	8
Voltage compliance	/	14 (±7) V	72 (±36) V
Inductive link parameters	*Transmitter coil:* Ø 50 mm 24 μH	*Receiver coil:* Ø 26.3 mm 47 μH	/
Communication	CAN bus, Bluetooth	Bluetooth	/

**Table 2:** Features and performances of the IPGs founded in the literature and the one presented in this work.

	D. Jiang, et al. [25]	V. Sivaji, et al. [26]	A. Deshmukh, et al. [27]	W. Kang, et al. [29]	This Work
Dimension	46 mm × 42.8 mm × 8.8 mm	13 mm × 8 mm × 3 mm	/	/	33.5 mm × 27.5 mm × 3.3 mm
Voltage compliance	18 V	12 V	16 V	28 V	14 V
Pulse amplitude	0–3 mA, step size 12 μA	0–1.2 mA, step size 20 μA	±500 μA up to ±4 mA	10 μA–2 mA. Step size 10 μA	±20 nA–±5 mA programmable
Pulse frequency	1–500 pps	10–30 Hz, step size 1 Hz	20 Hz	1–10 kHz	100–600 Hz * programmable
Pulse width	Cathodic phase: 0–500 μs, Anodic phase: 1–8 times cathodic width	100–250 μs, step size 10 μs	50 μs–1 ms	24–9999 μs	100–500 μs ** programmable
Num channels	3 × (6 active + 2 return)	2	2	4	3 × 10
Inductive link parameters	*Receiver coil:* 28 mm diameter, 0.5 mm gauge, 7 turns, 2.52 μH	*Receiver coil:* 9-turn 3-layer coil	/	*Receiver coil:* 34-AWG Teflon insulated wire–square 38 mm × 38 mm;	*Receiver coil:* 26.3 mm diameter-47 μH
Communication	9.6 MHz carrier frequency, 400 kb/s OOK downlink, 600 kb/s PPSK uplink	Inductive data link, Near field communication (NFC)	Wi-Fi radio operating at a frequency carrier of 2.4 GHz.	Wireless industrial scientific and medical (ISM) bands 2.4 GHz	Bluetooth 5.3 (2.4 GHz)

* Tested values, theoretical pulse frequency value is up to 20 kHz. ** Tested values, theoretical pulse width value is up to 15 min.

## Data Availability

The raw data supporting the conclusions of this article will be made available by the authors, without undue reservation.
